# His Majesty and the Hospitals

**Published:** 1902-05-24

**Authors:** 


### HIS MAJESTY AND THE HOSPITALS.

THE Prince of Wales' idea of a family gift to the hospitals on the King's birthday through King Edward's Hospital Fund is growing in popularity. The Lord Mayor has invited the Mayors throughout London and the country and every bank to receive subscriptions. On the King's birthday, Friday, May 30th, at every table throughout the country the head of the household will receive the voluntary offerings of members of his family and household and pay them into the nearest bank to the credit of King Edward's Hospital Fund. Thus the Coronation Gift to the King will include the gifts of the poorest and richest families in the land, and happily unite monarch and people in a free-will offering for the benefit of the suffering and the sick. The Book of the Coronation Gift, containing the name of every contributing family, should have an historical importance and interest.

